# Effects of Endosulfan on Predator–Prey Interactions Between Catfish and *Schistosoma* Host Snails

**DOI:** 10.1007/s00244-016-0275-7

**Published:** 2016-03-31

**Authors:** Concillia Monde, Stephen Syampungani, Paul J. Van den Brink

**Affiliations:** Department of Aquatic Ecology and Water Quality Management, Wageningen University and Research Centre, Wageningen University, P.O. Box 47, 6700 AA Wageningen, The Netherlands; Department of Zoology and Aquatic Sciences, Copperbelt University, Jambo Drive, Riverside, P.O. Box 21692, Kitwe, Zambia; Department of Plant and Environmental Sciences, Copperbelt University, Jambo Drive, Riverside, P.O Box 21692, Kitwe, Zambia; Alterra, Wageningen University and Research Centre, P.O. Box 47, 6700 AA Wageningen, The Netherlands

## Abstract

The effect of the pesticide endosulfan on predator–prey interactions between catfish and *Schistosoma* host snails was assessed in static tank experiments. Hybrid catfish (*Clarias gariepinus* × *C. ngamensis*) and *Bulinus globosus* were subjected to various endosulfan concentrations including an untreated control. The 48- and 96-h LC_50_ values for catfish were 1.0 and <0.5 µg/L, respectively, whereas the 48- and 96-h LC_50_ values for snails were 1137 and 810 µg/L. To assess sublethal effects on the feeding of the catfish on *B*. *globosus*, endosulfan concentrations between 0.03 and 1.0 µg/L were used. Predation was significantly greater (*p* < 0.001) in control tanks than in all other treatments. There was progressively decreasing predation with increasing toxicant concentration. Biological control of *Schistosoma* host snails using fish may be affected in endosulfan-polluted aquatic systems of Southern Africa because it has been found present at concentrations that are indicated to cause lethal effects on the evaluated hybrid catfish and to inhibit the predation of snails by this hybrid catfish.

**Electronic supplementary material:**

The online version of this article (doi:10.1007/s00244-016-0275-7) contains supplementary material, which is available to authorized users.

As the global human population continues to increase, the pressure on natural resources to provide goods and services also increases (Millenium Ecosystem Assessment 2005). This entails an increase in food production through agriculture. The use of chemicals is viewed as a panacea to improve the productivity of agriculture. As a result, the application of chemical fertilizers and pesticides to improve crop health and yield has increased worldwide. Global estimates of pesticide use in 2006 and 2007 are at approximately 2.4 million tonnes of active ingredients/year (United States Environmental Protection Agency 2011). However, not all of the pesticides applied will reach the targeted organisms. Estimates show that only approximately 0.3 % of pesticides applied reach the target organisms, whereas 99.7 % contaminates the surrounding environmental compartments, such as air, soil, and water, through spray drift, leaching, and runoff (van der Werf 1996). Pesticides may have direct and indirect effects on nontarget organisms in both terrestrial and aquatic ecosystems. Fish kills and alteration in the structure of invertebrate communities have been reported as well as effects on higher predators such as birds (Schäfer et al. 2011).

The majority of studies investigating the effects of pesticides on nontarget organisms have focused on their effect on individuals or single species. Several studies on how pollutants affect fish behaviour have been previously reviewed (Weis and Candelmo 2012). However, to understand the effects of these pollutants on interspecific interactions, such as predation, predators, and their prey, should be studied simultaneously to mimic natural conditions (Junges et al. 2010). Few studies focusing on the effect of pesticides on predator–prey interactions have been reported. Results from these studies have shown that different pesticides can disrupt intraspecific and interspecific interactions between organisms and therefore alter the ecological functioning of ecosystems (Bridges 1999; Relyea and Hoverman 2006; Junges et al. 2010). This could occur by causing mortality of either the predator or prey or by interfering with their physiology. However, there is no evidence of studies in the literature looking specifically at the effect of pesticides on snail predation by fish. Organophosphate, carbamate, and organochlorine pesticides affect the nervous system of vertebrates by inhibiting the enzyme cholinesterase, which regulates acetylcholine, a neurotransmitter that is important for nerve function (DeLorenzo et al. 2001). Boone and Semlitsch (2003) observed increased survival of bullfrogs *Rana catesbeiana* after the extermination of predatory crayfish *Orconectes* sp. and bluegill sunfish *Lepomis macrochirus* in a pond experiment. The death of the predators was due to toxicity of the carbamate insecticide carbaryl. *Micropterus salmoides* exposed to the organochlorine pesticide pentachlorophenol (67–88 µg PCP/L) showed decreased feeding capacity, made more mistakes by aiming at nonprey items and failed to capture its targets more often than the unexposed fish (Mathers et al. 1985; Brown et al. 1987). Cessation of all locomotion, inability to maintain position, and decreased feeding in *Oncorhynchus kisutch* exposed to organophosphate insecticide fenitrothion was reported by Bull and McInerney (1974). Increased prey survival was the outcome of predator–prey interactions of *Synbranchus marmoratus* and *Hypsiboas pulchellu*s exposed to 2500 μg/L of fenitrothion. Fenitrothion toxicity appeared to have modified prey behaviour by making them less mobile and hence less visible to the predator (Junges et al. 2010).

Agriculture makes an important contribution to livelihoods and economies of southern African countries. It contributes approximately 8 % to the gross domestic product of southern Africa (Chilonda and Minde 2007), and >80 % of the populations of Malawi, Zambia, and Mozambique depend on agriculture for subsistence (Mucavele 2013). As a coping strategy for food production and income generation, wetlands and uplands are used in an integrated manner by the rural people to achieve sustained livelihoods. Agricultural fertilizers and pesticides are used in these farming systems to boost crop yields. Unfortunately, like in many developing countries, banned, nonpatented, obsolete, and environmentally persistent pesticides are widely used in southern African countries including Zambia, Zimbabwe, and South Africa (Stockholm Convention on Persistent Organic Pollutants Review Committee 2010). However, in many of these countries studies to quantify the effects of these pollutants on the environment have rarely been performed. One such pesticide with persistent effects to the environment still in use in southern African is endosulfan (Deedat et al. 1997; Stockholm Convention on Persistent Organic Pollutants Review Committee 2010).

Endosulfan is an organochlorine insecticide that is hazardous to the environment. It is composed of two isomers, a- and b-endosulfan, which degrade into endosulfan sulphate, and it is persistent, nonbiodegradable and capable of biomagnification as it moves up the food chain (Agbohessi et al. 2014). Endosulfan may enter the aquatic ecosystem through runoff, direct spray, leaching through the soil and volatilization into the atmosphere, and later as precipitation. Although monitoring data for pesticides is very difficult to find for developing countries (Van Dyk and Pletschke 2011), endosulfan has been found in water and sediment of many water bodies in Africa (Nyangababo et al. 2005; Syakalima et al. 2006; Ezemonye et al. 2010; El Bouraie et al. 2011; Ansara-Ross et al. 2012; Ibigbami et al. 2015) (Supplementary Information Table S1).

Many studies have documented the toxicity of endosulfan to aquatic organisms including tadpoles, snails, and fish (Ellis-Tabanor and Hyslop 2005; Jones et al. 2009; Agbohessi et al. 2014). These effects can be direct on the organism’s health by affecting the animal’s physiological function or indirect by affecting trophic interactions such as competition and predation (Schäfer et al. 2011). Although it has been found not to be as persistent in tropical climates as in temperate climates (Mwangala et al. 1997), endosulfan toxicity to aquatic organisms may have a bearing on the functioning of aquatic interactions in the tropics because it is found at concentrations that are likely to affect arthropods, invertebrates, and fish (Hose and Van den Brink 2004).

To date, despite the fact that many studies having documented the effects of pesticides on aquatic invertebrate and vertebrate fauna, none of these studies have assessed the effect of pesticides on the predator–prey interaction of *Schistosoma* host snails and their fish predators. However, studies specifically looking at the effects of pesticides on host snails have mainly focused on organophosphate pesticides, including the insecticides profenophos and chlorpyrifos, as studied by Hasheesh and Mohamed (2011) and Mohamed (2011), and these were found to have molluscicidal effects. Ibrahim et al. (1992) observed decreased egg production and egg hatchability at sublethal concentrations and mortality at greater concentrations of chlorpyrifos by *Biomphalaria alexandrina*. The effect of organochlorine pesticides on host snails is largely unknown. Similarly, several studies have documented the effect of pesticides on fish. These include Bacchetta et al. (2014), who observed deleterious effects of endosulfan and lambda-cyhalothrin on *Piaractus mesopotamicus.* Refer to Bacchetta et al. (2011) and Napit (2013) for more studies on the effects of various pesticides on different species of fish. Hence, the present study investigated the effect of an organochlorine pesticide endosulfan on the predator–prey interactions of hybrid catfish and *Bulinus globosus* snails using environmentally relevant concentrations. In doing so we were specifically seeking to address two objectives: (1) to determine what pesticide concentration would impact both the host snails and the hybrid catfish; and (2) to investigate whether sublethal pesticide concentrations have an effect on the predation efficiency of catfish (*Clarias gariepinus/C. ngamensis* hybrid).

## Materials and Methods

### Test Animals

Hybrid African catfish were collected from outdoor ponds at the Aquaculture Development and Research Centre in Mwekera, Zambia. The ponds were drained, and a scoop net (made of 2 × 2-mm mesh size sieve that was supported by a metal frame mounted on a 1.5-m wooden handle) was used. One hundred twenty-four fish of weight, ranging in weight from 180 to 310 g, were kept in plastic tanks (100 L) at a stocking density of two fish/tank due to their aggressive behaviour. The fish were left to acclimatize for 5 days, during which time they were fed commercial fish meal (pellets of 30 % crude proteins, 12 % crude fat) twice a day at 4 % body weight. To decrease the accumulation of excess food and fish faeces, water in the tanks was changed every other day by siphoning 50 % of the spent water and filling with fresh borehole water.

One thousand snails (*Bulinus globosus*) were collected from ponds at NADRC. These were picked by gloved hands and kept in plastic tanks. Raw lettuce was provided as feed for snails ad libitum.

### Test Water Parameters

We monitored three water-quality parameters—temperature, pH, and dissolved oxygen—for the predation experiment using an AM-200 Aquaread GPS Aquameter. All tests were performed under static conditions (i.e., the test water was not changed for the duration of the experiment). This was important to capture the short-term peak concentration effects rather than the long-term, chronic-exposure effects of endosulfan because it is known not to be persistent in tropical environments and because applications of pesticides are usually many months apart.

### Endosulfan Preparation and Analysis

The insecticide endosulfan was chosen because it is one of the commonly used pesticides for both seasonal and off-season farming in Zambia. Vegetable gardening is an off-season activity in most parts of the country and is performed on the banks of rivers, streams, and other water impoundments. Sionex 35 EC was purchased from VINCO Agrochemicals in Zambia. The concentration of the active ingredient (endosulfan) in the Sionex 35 EC was 350 g/L, on which all dilutions to make the test solutions were based. A stock solution of 10 mg/L was made from the original concentration. Desired test solutions were obtained by diluting various amounts of the stock solution in 500 L of borehole water (Table 1). All test solutions were freshly prepared before commencement of the tests.

**Table 1:** Nominal test concentration and subsequent HPLC-measured concentrations at the start and end of experimentation

Experiment	Nominal concentration (µg/L)	Measured concentration at start (µg/L)	Measured concentration at end (µg/L)
Effect on feeding	0.03	0.033	0.028
	0.1	0.17	0.096
	0.3	0.33	0.31
	1.0	1.3	1.1
Effect on catfish	0.5	0.46	0.39
	1.0	1.2	1.2
	1.1	1.3	1.2
	1.2	1.5	1.4
Effect on snails	100	101	100
	200	267	212
	400	399	365
	500	451	427
	1000	997	982
	1200	1210	1180

Analytical verification of pesticide concentration in the test water was performed through high-performance liquid chromatography (HPLC)-ultraviolet (UV) procedure before and after experimentation. For the extraction of pesticide residues from water, the liquid–liquid extraction method was adopted. We collected water samples at the beginning and at the end of each experiment from the test water into 500-mL amber-glass bottles. Ethyl acetate was used to extract endosulfan from the water at a ratio of 2:1 (sample to solvent). The sample–solvent mixture was then shaken with a manual shaker for 30 min to extract the endosulfan to the organic solvent. The mixture was transferred into separatory funnels and allowed to stand for 20 min to allow separation of the organic layer from the water layer. The organic layer was collected into 100-mL amber glass bottles and sealed with glass tops. Before analysis by HPLC–UV, the samples were further dried using anhydrous sodium sulphate. HPLC operating conditions for the analysis were as follow: The mobile phase was acetonitrile and water (70:30) using an isocratic elution. Injection volume of the sample was 10 μL with a flow rate of 1.0 mL/min. The oven temperature was 40 °C, and the run time was 20 min. The column used was a Shim-pack VP-ODS; 250 × 4.6-mm I.D, and the UV detector used ran at 254 nm. The used standard was endosulfan (mixed isomers) CAS no. 115-29-7 with a purity of 100 %. The actual concentration of the standard was 1002 μg/mL or 0.1 % in methanol solvent. This standard was purchased from Accu Standard, Inc.

### Experimental Design

Two sets of tank experiments were set up to (1) determine effect of endosulfan exposure on snail and catfish survival; and (2) determine effect of sublethal endosulfan exposure on host snail predation by catfish.

### Observable Behavioural Responses

Changes in fish behaviour were qualitatively quantified as either normal (−), low (+), moderate (++), or severe (+++). The observations were terminated after 72 h because no catfish survived beyond 72 h at all concentrations except in the control arm.

### Effect on Catfish and Snail Survival

In the toxicity experiment with hybrid catfish, 20 tanks of 200 L were filled with 50 L of endosulfan-treated water. Five concentrations (0, 0.5, 1.0, 1.5, and 2.0 µg/L) were tested in the range-finding test, which lasted for 24 h. Because mortality was observed in a few hours in the two highest test concentrations, we decided to use five concentrations of 0, 0.5, 1.0, 1.1, and 1.2 µg/L to test for catfish survival to technical endosulfan toxicity in the final test. These concentrations were selected based on the results of a range-finding test, which itself was based on the species sensitivity distribution and the resulting hazardous concentration 5 % value for fish of 0.31 µg/L as reported by Hose and Van den Brink (2004). Juvenile catfish of weight 190–250 g were stocked with five in each tank. All concentrations were replicated four times. Observations started immediately after the fish were exposed to endosulfan and continued every 12 h. In the experiment for endosulfan toxicity to snails, 28 tanks of 50 L were used. Seven pesticide concentrations of 0, 100, 200, 400, 500, 1000, and 1200 µg/L were prepared based on the LC_50_ values provided by Yasser et al. (2008) for other gastropods and a range-finding test involving 10 (0, 200, 400, 600, 800, 1000, 1200, 1400, 1600, and 1800 µg/L) concentrations. In the range-finding test, we observed >50 % mortality in the 1400 to 1800 µg/L concentrations during the 24-h observation time. All definitive test concentrations were evaluated in four replicates. Twenty-eight groups of 20 snails (9- to 15-mm size) were each randomly stocked in the prepared experimental units. Observations started immediately after the snails were exposed to the endosulfan-treated water and continued every 24 h for 96 h. All tanks were covered with netting material to prevent test organisms from escaping. A completely randomized design was used to perform these experiments. The end points for both hybrid catfish and *B. globosus* was death of exposed individuals, which was recorded on a daily basis. Fish were considered dead when they were found either floating on the surface of the water upside down or lying motionless at the bottom even when provoked. Snails were considered dead when they did not respond to pricking of their soft parts with a sharp object. All dead specimens were removed from the experimental units.

### Effect on Snail Predation by Catfish

To evaluate the sublethal effect of endosulfan, predation of snails by hybrid catfish was evaluated in 20 tanks of 50 L by stocking each tank with 50 *B. globosus* of sizes ranging between 8- to 15-mm shell height. The tanks were filled with 20 L of water with one of the endosulfan test concentrations. The control tanks received water with no endosulfan, whereas the other tanks received water with 0.03, 0.1, 0.3, or 1.0 µg/L concentrations; each was replicated four times. These concentrations were selected based on the results of the lethality test, which yielded 48-h LC_10_ values of approximately 1 µg/L. The LC_10_ was preferred over the LC_50_ to decrease the chances of mortality in the sublethal tests. A single catfish was introduced into every tank, and observations were commenced 24 h later and lasted for 96 h. All of the catfish used fell within a size range of 300 ± 15 g and were starved for 24 h before the start of the experiment. Snails continued receiving 2 g of raw lettuce per tank ad libitum, and the medium was not renewed for the whole duration of the experiment. The effect of endosulfan on the snail predation efficiency of hybrid catfish was measured through the number of snails eaten per day compared with the control. A snail was considered eaten only when it was missing from the tank.

### Statistical Analysis

The LC_10_, LC_50_, and LC_90_ values of the toxicity experiments were calculated by means of log-logistic regression using the software GenStat 11th (VSN International Ltd., Oxford, UK) according to Rubach et al. (2011). Because we used a static bioassay, LC_10_, LC_50_, and LC_90_ values were calculated using the geometric means of the start and end concentrations for catfish and *B. globosus* at each sampling period. The effect of endosulfan toxicity on predator–prey interactions of catfish and snails was assessed by comparing the number of eaten snails at different exposure concentration over time through regression analysis. Generalized linear models (GLMs) were used to assess the significance of the differences among treatments for each sampling period. The model used for the GLM analysis was adapted to the data distribution of the measured end points. Mortality was assessed using a binomial distribution and logit as the link function. The effects of the pesticide concentration on the evaluated end points were considered to be significant when the calculated *p* values were <0.05.

## Results

### Test Water Parameters

There were no large differences between the nominal and measured concentrations (Table 1). The concentrations were on average 14 % (±20 %) greater than the nominal concentrations. Endosulfan did not show a clear dissipation rate for the three experiments. An average dissipation of 8.1 % (±4.8 %) was found in the 2 days and of 6.6 % (±7.4 %) in the 4 days for the lethal-effects experiment on catfish and snails, respectively, whereas a dissipation of 20 % (±16 %) was found in the 4-day experiment evaluating the sublethal effects of endosulfan on predator–prey interactions (Table 1).

The levels of the three water-quality parameters (temperature, dissolved oxygen, and pH) monitored in the experimental units over time during the predation experiment are shown in Figure S1 of the Supplementary Information. There were fluctuations in these parameters over the duration of the experiments. Temperature ranged between 23.0 and 22.7 °C, pH between 4.15 and 5.70, and DO between 3.5 and 6.7 mg/L. There was no significant difference (*p* = 0.892) in the temperature between the treatments and the control. However, significant differences were observed in pH (*p* = 0.002) and DO (*p* < 0.001). The control units (0.0 µg/L) had the highest DO, whereas the highest endosulfan concentration (1.0 µg/L) units had the lowest. The pH reached its lowest (4.15) in the highest endosulfan concentration at 96 h (Fig. S1).

### Observable Behavioural Responses

Five responses were observed in fish exposed to endosulfan. These include increased or erratic swimming, attempts to jump out of the tanks, gasping for air, disorientation, and secretion of thick layer of mucus on the body. The intensity of these responses were dependent on endosulfan concentration. Fish in the 0.5 and 1.0 µg/L concentrations exhibited severe jumping and swimming on exposure to endosulfan, whereas these the frequency of traits was low for fish exposed to greater concentrations. Fish exposed to greater concentrations showed more signs of exhaustion, gasping for air, and disorientation. All fish exposed to endosulfan secreted thick masses of mucus on their bodies (Table S2).

### Effect on Catfish and Snail Survival

For catfish, survival in the control remained at 95 % at both 24 and 48 h (Table S3). At the lowest endosulfan concentration, survival was equally high, i.e., 90 % for 24 and 48 h, respectively. However, marked decreases in survival rates were recorded for the concentrations ranging between 1.0 and 1.2 µg/L for both days. Only 55 and 45 % survival was found in the 1.0 µg/L concentration, 5 and 0 % in the 1.1 µg/L concentration, and 0 % at the highest concentration (1.2 µg/L) at both 24 and 48 h, respectively (Table S3). At 72 h, all of the catfish exposed to endosulfan toxicity were dead leaving only those in the control tanks alive. Table 2 lists the associated LC_10_, LC_50,_ and LC_90_ values for the catfish, which are all close to 1 µg/L.

**Table 2:** LC values (μg/L) and their confidence intervals at the various time points

Experiment	End point	Time			
		24 h	48 h	72 h	96 h
Catfish	LC_10_	0.948 (0.896–1.003)	0.988 (0.978–0.998)	<0.5^a^	<0.5^a^
	LC_50_	1.009 (0.983–1.036)	1.000 (0.995–1.005)	<0.5^a^	<0.5^a^
	LC_90_	1.075 (1.023–1.13)	1.011 (1.003–1.02)	<0.5^a^	<0.5^a^
*B. globosus*	LC_10_	807 (551–1181)	682 (485–959)	372 (257–540)	485 (336–701)
	LC_50_	4160 (1336–12,956)	1137 (1041–1245)	907 (787–1044)	810 (690–950)
	LC_90_	21,457 (2056–223,941)	1902 (1306–2769)	2209 (1585–3077)	1351 (1139–1602)

^a^No catfish survived beyond 72 h except in the controls

During the first 24 h, *B. globosus* survival ranged between 80 and 100 % for all treatments <1000 µg/L and between 67 and 76 % for the treatments ≥1000 µg/L (Table S4). However, at 48 h survival in the control and at concentrations <1000 µg/L decreased to between 67 and 82 %, whereas for the concentrations 1000 and 1200 µg/L survival decreased to 59 and 39 %, respectively. At 96 h, only 20 and 11 % *B. globosus* were still surviving at the two highest concentrations, respectively, whereas survival remained >58 % at the lower concentrations during the same period (Table S4). Table 2 lists the associated LC_10_, LC _50_, and LC_90_ values for *B. globosus*, which are much greater than those found for the catfish.

### Effect on Snail Predation by Catfish

**Fig. 1:**
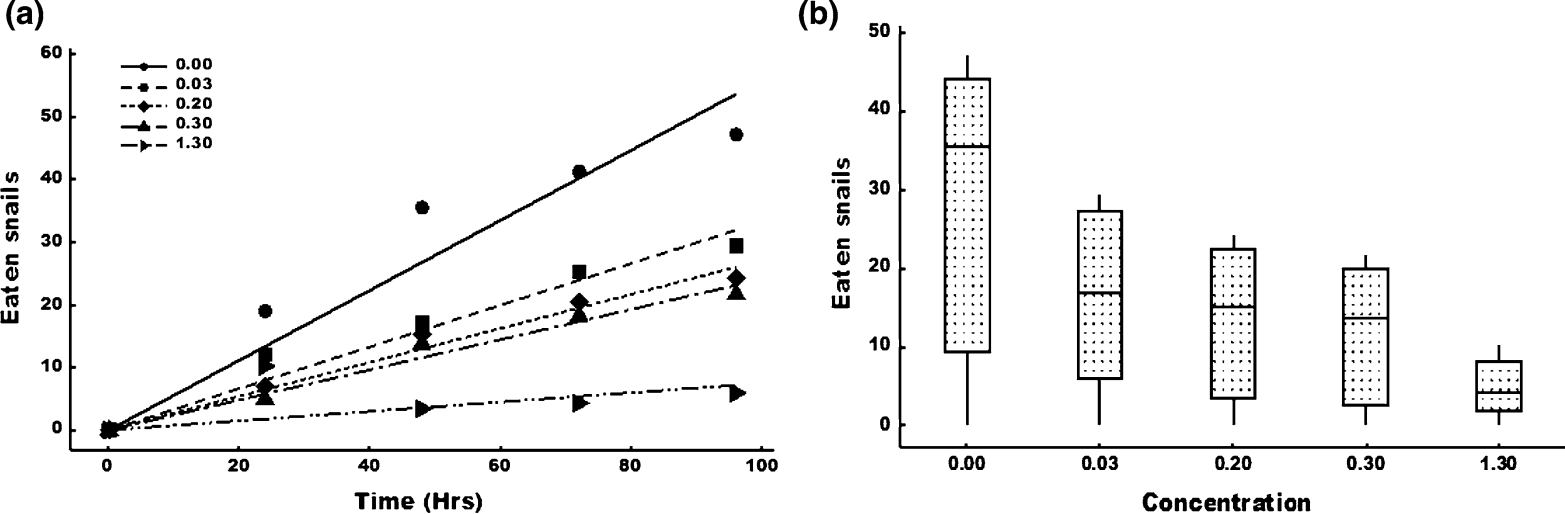
Average of the cumulative number of snails eaten by the catfish per treatment level over time and **a** the associated GLM fits and **b** variation in the cumulative number of snails eaten at the different endosulfan concentrations at the end of the experiment

Exposure to sublethal concentrations of endosulfan resulted in significant differences in catfish rate of predation. At the end of the 4-day observation period, >7 times more snails had been eaten in the controls than in the high-endosulfan concentration (1.30 µg/L) (Fig. 1a). There were catfish deaths in the 0.3 and 1.2 µg/L treatments, and replacements were made immediately (Table S5).

GLM tests yielded significant differences in snail predation (*p* < 0.001 for all sampling dates). The number of snails consumed per day was dependent on pesticide exposure regimes with predation being significantly greater in the controls than in all other treatments (Fig. 1b).

## Discussion

### Test Water Parameters

The aim of this study was to assess the toxic effect of endosulfan on both the predator hybrid catfish and the prey *B. globosus* as well as on the predation efficiency of the hybrid catfish. Endosulfan toxicity to aquatic organisms, as reported in many studies, was also shown in the present study. The decrease in the levels of pH and DO observed in this study in the highest treatment level of the predation experiment (Fig. S1) show the way fish are affected by the toxicant. Endosulfan inhibits the action of the neurotransmitter gamma-aminobutyric acid, which leads to a state of uncontrolled neuronal excitation (Rozman and Klaassen 2007). Interference in the activity of Ca^2+^-ATPase and hence calcium transportation, as well as phosphokinase activities, may also be induced by exposure to endosulfan (World Health Organization 2000). Matthiessen and Roberts (1982) reported hyperactivity in *Tilapia rendalli* exposed to endosulfan. This behavioural response may have caused an increase in the rate of respiration and hence a decrease in levels of DO and an increase in dissolved carbon dioxide in the water in the present study. Muthukumar et al. (2009) observed a rapid increase in opercular movements of *Tilapia mossambicus* exposed to sublethal concentrations of endosulfan as a way of increasing oxygen uptake to mitigate the stress caused by the toxicant. Dissolved carbon dioxide reacts with water to form carbonic acid, which in turn decreases the pH (Wurts and Durborow 1992). In the present study, although there was a significant decrease in DO in the highest endosulfan concentration, it remained within the tolerable range for catfish (Mallya 2007). The pH observed in this experiment (4.15–5.70) was in some cases not optimal for fish, which according to Alabaster and Lloyd (2013) decreases between 5 and 9. Although fish may survive at lower pH ranges as was the case was in our study, acidic conditions may affect the fish’s endocrine system, break down the gill structure, or even suffocate the fish due to excessive accumulation of mucus (Kwong et al. 2014).

### Behavioural Responses and Effect on Catfish Survival

In the toxicity test, the hybrid catfish exhibited a number of behavioural responses on exposure to endosulfan at all concentrations. These included increased swimming activities, attempts to jump out of the tanks, violent and erratic swimming, gasping for air, disorientation, and secretion of thick masses of mucus from their bodies. Capkin et al. (2006) reported similar behavioural changes for rainbow trout (*Oncorhynchus mykiss*) exposed to endosulfan. These behavioural changes may be an indication of the effect of endosulfan on the fish nervous system. Endosulfan is highly toxic to fish and other aquatic organisms, but its toxicity varies among different species. The 48-h LC_50_ value for hybrid catfish found in this study is 1.0 μg/L. The LC_10_ and LC_90_ values are also quite close to 1.0 μg/L (Table 2) implying that this species has a low intraspecific variation in sensitivity to endosulfan toxicity. However, the death of catfish occurred within 1 h of exposure in the 1.2 μg/L experimental units, and all except those in the control died between 48 and 72 h. Catfish are known to rapidly accumulate high levels of endosulfan in their body tissues. Zeid et al. (2005) reported increased levels of endosulfan in juvenile African catfish after 6 h of exposure. Jenyo-Oni et al. (2011) recorded a 48-h LC_50_ value of 4.68 μg/L, whereas Yekeen and Fawole (2011) reported a 96-h LC_50_ value of 52 μg/L for juvenile *C. gariepinus.* Our finding are similar to those for several other species of fish of the Okavango Delta in Botswana ranging from 1.2 to 7.4 μg/L (Fox and Matthiessen 1982). The differences in LC_50_ values observed in many studies could be attributed to differences in the length of observation, age and species of fish used, and, in the case of this experiment, the use of a hybrid fish compared with pure breeds used in other experiments. From the results of this toxicity experiment, we only expected effects of endosulfan on fish survival at the highest concentration of the predation experiment.

### Effect on Snail Survival

For *B. globosus*, we found a 96-h LC_50_ value of 810 μg/L, so we did not expect any direct effect of endosulfan on snail survival in the predation experiment. The control mortality of the experiment was, however, rather high (Table S4). This could have been a result of stress due to the long period the animals were kept under artificial conditions in the laboratory before experimentation. Our finding is within the range of values obtained by other studies involving the acute toxicity of endosulfan to freshwater snails. Yasser et al. (2008) performed a toxicity test with *Lymnaea radix* and found 96-h LC_50_ values of 380 and 910 μg/L for juveniles and adults, respectively. Jonnalagadda and Rao (1996) performed a test with *Bellamya disimilis* and recorded a 96-h LC_50_ value of 1800 μg/L, whereas Oliveira-Filho et al. (2005) found 96-h LC_50_ values of 120 and 890 μg/L for *Biomphalaria tenagophila* juveniles and adults, respectively. Greater values were reported, however, for three freshwater snails by Ellis-Tabanor and Hyslop (2007), who obtained 96-h LC_50_ values of 2300, 1740, and 1350 μg/L for *Melanoides tuberculata*, *Thiara granifera*, and *Planorbella duryi*, respectively, whereas Otludil et al. (2004) reported an LC_50_ value of 3230 μg/L for *Planorbarius corneus*.

### Effect on Snail Predation by Catfish

The sublethal effects were assessed by monitoring the changes in the catfish rate of predation of *B. globosus*. The effect of endosulfan on predation was dose and time dependent (Fig. 1). However, mortality of some predators was observed in the highest concentration (Table S4). Replacement of the dead individuals with new previously unexposed individuals may have resulted in underestimation of the inhibition on predation in the high concentration treatments. Due to its effect on the nervous system (Ellis-Tabanor and Hyslop 2005) and enzyme activity (Tripathi and Verma 2004), endosulfan affects physiological and metabolic functions in organisms. One such effect is the inhibition of the activity of phagocytic cells, which renders the organism less able to defend itself against disease. Girón-Pérez et al. (2008), in a study to determine the effect of endosulfan on the phagocytic activity in *Oreochromis niloticus*, focused on two parameters—the phagocytic index and the percentage of active cells—and found that endosulfan had a significantly negative effect on both parameters. Blood is a reflector of the pathological and physiological status of the body of an organism, and hence it shows the structural and functional well-being of the organism. The decrease in feeding observed in this study may be a reflection of the adverse effect that endosulfan may have exerted on catfish haematological parameters. Several studies have reported the effects of endosulfan on haematological parameters of aquatic organisms. For instance, Jenkins et al. (2003) observed a decrease in haematological parameters—such as erythrocyte counts, haemoglobin percentage, and haematocrit values—in *Cyprinus carpio* exposed to endosulfan, and the effects were dose dependent. Ndimele et al. (2015) reported microcytic hypochromic anaemia in fingerlings of catfish (*C. gariepinus*) after 24 h of exposure to endosulfan. A decrease in haemoglobin has a direct effect on the amount of oxygen available to an organism. In our experiment, the decreased feeding may have been a coping strategy to depressed oxygen supply to the various organs of the catfish because the fish’s affinity for oxygen decreased due to endosulfan toxicity.

The inhibitory effect of endosulfan on fish’s acetylcholinesterase (AChE) may also have affected interspecific relations between the catfish and the snails. AChE is an important enzyme catalyzing the activities of acetylcholine, which is a neurotransmitter responsible for psychomotor activities of fish. This inhibitory effect was observed in *Lepomis macrochirus, Labeo rohita, Danio rerio,* and *Jenynsia multidentata* exposed to endosulfan (Dutta and Arends 2003; Ballesteros et al. 2009; Kumar et al. 2012; Pereira et al. 2012). These alterations in fish behaviour have an effect on their ecological functions such as feeding, predator avoidance, foraging, and reproduction hence their survival (Banaee 2012).

### Consequences for the Prevalence of Schistosomiasis

From this study, it is clear that endosulfan pollution has a negative affect the predator–prey interactions of *Schistosoma* host snails and their fish predators. This effect appears to favour the survival of the host snails over their predators. This finding has further augmented the claim that endosulfan is generally less toxic to nonarthropod invertebrate taxa than it is to fish (Hose and Van den Brink 2004). The decreased predation pressure of the catfish toward their prey due to pesticide pollution has the potential to foster rapid population growth in the prey population. This increase may make it easier for *Schistosoma* to encounter and infect the snails. Endosulfan is present in water and sediment samples taken in Africa (Table S1) at concentrations capable of eliciting both sublethal and lethal effects on catfish, which may decrease the effectiveness of biocontrol using hybrid catfish. The implications of this finding to biological control of schistosomiasis is that in places where pesticides, such as endosulfan, are used adjacent to transmission sites fish may not be effective control agents. Although fish may be affected at very low doses, snails remain unaffected by these concentrations.

Because agriculture is an important economic activity in most poor countries (Dao 2012), a shift toward using pesticides that are less lethal to snail predators would be an important factor in balancing between agricultural productivity and decreased prevalence of schistosomiasis in endemic areas. In addition to endosulfan, several insecticides are found in aquatic ecosystems in many African countries including South Africa (Quinn et al. 2011), Mali (Dem et al. 2007), Nigeria (Ibigbami et al. 2015; Ogbeide et al. 2015), and Zambia (Syakalima et al. 2006). Evaluation of the effects of these pesticides and their combinations on predator–prey interactions of host snails and their predators should be considered.

## Electronic supplementary material

Below is the link to the electronic supplementary material.

Supplementary material 1 (DOCX 86 kb)

## References

[CR1] Agbohessi TP, Toko II, N’tcha I, Geay F, Mandiki S, Kestemont P (2014). Exposure to agricultural pesticides impairs growth, feed utilization and energy budget in African Catfish *Clarias gariepinus* (Burchell, 1822) fingerlings. Int Aquat Res.

[CR2] Alabaster JS, Lloyd RS (2013). Water quality criteria for freshwater fish.

[CR3] Ansara-Ross T, Wepener V, Van den Brink P, Ross M (2012). Pesticides in South African fresh waters. Afr J Aquat Sci.

[CR4] Bacchetta C, Cazenave J, Parma MJ, Biancucci GF (2011). Biochemical stress responses in tissues of the cichlid fish *Cichlasoma dimerus* exposed to a commercial formulation of endosulfan. Arch Environ Contam Toxicol.

[CR5] Bacchetta C, Rossi A, Ale A, Campana M, Parma MJ, Cazenave J (2014). Combined toxicological effects of pesticides: a fish multi-biomarker approach. Ecol Indic.

[CR6] Ballesteros M, Durando P, Nores M, Díaz M, Bistoni M, Wunderlin D (2009). Endosulfan induces changes in spontaneous swimming activity and acetylcholinesterase activity of *Jenynsia multidentata* (Anablepidae, Cyprinodontiformes). Environ Pollut.

[CR7] Banaee M (2012) Adverse effect of insecticides on various aspects of fish’s biology and physiology. Volume. INTECH Open Access Publisher

[CR9] Boone MD, Semlitsch RD (2003). Interactions of bullfrog tadpole predators and an insecticide: predation release and facilitation. Oecologia.

[CR10] Bridges CM (1999). Predator-prey interactions between two amphibian species: effects of insecticide exposure. Aquat Ecol.

[CR11] Brown JA, Johansen PH, Colgan PW, Mathers RA (1987). Impairment of early feeding behavior of largemouth bass by pentachlorophenol exposure: a preliminary assessment. Trans Am Fish Soc.

[CR12] Bull CJ, McInerney JE (1974). Behavior of juvenile coho salmon (*Oncorhynchus kisutch*) exposed to Sumithion (fenitrothion), an organophosphate insecticide. J Fish Board Can.

[CR13] Capkin E, Altinok I, Karahan S (2006). Water quality and fish size affect toxicity of endosulfan, an organochlorine pesticide, to rainbow trout. Chemosphere.

[CR14] Chilonda P, Minde I (2007). Agricultural growth trends in Southern Africa.

[CR15] Dao MQ (2012). Population and economic growth in developing countries. Int J Acad Res Bus Soc Sci.

[CR16] Deedat YD, Maimbo GC, Phiri AS (1997) Effects of endosulfan on a maize agro-ecosystem in Zambia. Organochlor Insectic Afr Agroecosyst. 28(12):205–212

[CR17] DeLorenzo ME, Scott GI, Ross PE (2001). Toxicity of pesticides to aquatic microorganisms: a review. Environ Toxicol Chem.

[CR18] Dem S, Cobb J, Mullins D (2007). Pesticide residues in soil and water from four cotton growing areas of Mali, West Africa. J Agric Food Environ Sci.

[CR19] Dutta HM, Arends DA (2003). Effects of endosulfan on brain acetylcholinesterase activity in juvenile bluegill sunfish. Environ Res.

[CR20] El Bouraie MM, El Barbary A, Yehia M (2011). Determination of organochlorine pesticide (OCPs) in shallow observation wells from El-Rahawy contaminated area, Egypt. Environ Res Eng Manag.

[CR21] Ellis-Tabanor M, Hyslop E (2005). Effect of sublethal concentrations of endosulfan on growth and fecundity of two species of snails. Bull Environ Contam Toxicol.

[CR22] Ellis-Tabanor M, Hyslop EJ (2007). Acute toxicity of endosulfan to three freshwater snails in Jamaica. Carrib J Sci.

[CR23] Ezemonye LI, Ikpesu TO, Tongo I (2010). Distribution of endosulfan in water, sediment and fish from Warri river, Niger delta, Nigeria. Afr J Ecol.

[CR24] Fox P, Matthiessen P (1982). Acute toxicity to fish of low-dose aerosol applications of endosulfan to control tsetse fly in the Okavango Delta, Botswana. Environ Pollut A Ecol Biol.

[CR25] Girón-Pérez M, Montes-López M, García-Ramírez L, Romero-Bañuelos C, RobledoMarenco ML (2008). Effect of sub-lethal concentrations of endosulfan on phagocytic and hematological parameters in Nile tilapia (*Oreochromis niloticus*). Bull Environ Contam Toxicol.

[CR26] Hasheesh WS, Mohamed RT (2011). Bioassay of two pesticides on *Bulinus truncatus* snails with emphasis on some biological and histological parameters. Pestic Biochem Physiol.

[CR27] Hose G, Van den Brink P (2004). Confirming the species-sensitivity distribution concept for endosulfan using laboratory, mesocosm, and field data. Arch Environ Contam Toxicol.

[CR28] Ibigbami OA, Aiyesanmi AF, Adeyeye EI, Adebayo AO (2015). Persistent organochlorine pesticide residues in water, sediments and fish samples from Ogbese River. Environ Natl Resour Res.

[CR29] Ibrahim W, Furu P, Ibrahim A, Christensen N (1992). Effect of the organophosphorous insecticide, chlorpyrifos (Dursban), on growth, fecundity and mortality of *Biomphalaria alexandrina* and on the production of *Schistosoma mansoni* cercariae in the snail. J Helminthol.

[CR30] Jenkins F, Smith J, Rajanna B, Shameem U, Umadevi K, Sandhya V (2003). Effect of sub-lethal concentrations of endosulfan on hematological and serum biochemical parameters in the carp *Cyprinus carpio*. Bull Environ Contam Toxicol.

[CR31] Jenyo-Oni A, Ndimele P, Onuoha S (2011). Acute toxic effects of endosulfan (organochlorine pesticide) to fingerlings of African catfish (*Clarias gariepinus* Burchell, 1822). Am Eurasian J Agric Environ Sci.

[CR32] Jones DK, Hammond JI, Relyea RA (2009). Very highly toxic effects of endosulfan across nine species of tadpoles: lag effects and family-level sensitivity. Environ Toxicol Chem.

[CR33] Jonnalagadda P, Rao MB (1996). Histopathological changes induced by specific pesticides on some tissues of the fresh water snail, Bellamya dissimilis Müller. Bull Environ Contam Toxicol.

[CR34] Junges CM, Lajmanovich RC, Peltzer PM, Attademo AM, Bassó A (2010). Predator–prey interactions between *Synbranchus marmoratus* (Teleostei: synbranchidae) and *Hypsiboas pulchellus* tadpoles (Amphibia: Hylidae): importance of lateral line in nocturnal predation and effects of fenitrothion exposure. Chemosphere.

[CR35] Kumar N, Jadhao S, Chandan N, Kumar K, Jha A, Bhushan S (2012). Dietary choline, betaine and lecithin mitigates endosulfan-induced stress in *Labeo rohita* fingerlings. Fish Physiol Biochem.

[CR36] Kwong RW, Kumai Y, Perry SF (2014). The physiology of fish at low pH: the zebrafish as a model system. J Exp Biol.

[CR37] Mallya YJ (2007) The effects of dissolved oxygen on fish growth in aquaculture. The United Nations University Fisheries Training Programme, Final Project

[CR38] Mathers RA, Brown JA, Johansen PH (1985). The growth and feeding behaviour responses of largemouth bass (*Micropterus salmoides*) exposed to PCP. Aquat Toxicol.

[CR39] Matthiessen P, Roberts R (1982). Histopathological changes in the liver and brain of fish exposed to endosulfan insecticide during tsetse fly control operations in Botswana. J Fish Dis.

[CR40] Millenium Ecosystem Assessment (2005) Living beyond our means: natural assets and human well-being. Statement from the Board. UNEP, Paris, France

[CR41] Mohamed R (2011). Impact profenophos (pesticide) on infectivity of *Biomphalaria alexandrina* snails with *Schistosoma mansoni* miracidiaand on their physiological parameters. Open J Ecol.

[CR42] Mucavele FG (2013) True contribution of agriculture to economic growth and poverty reduction: Malawi, Mozambique and Zambia Synthesis Report

[CR43] Muthukumar G, Anbalagan R, Krishnan KR (2009). Adaptive changes in respiratory movements of an air breathing fish Tilapia mossambicus exposed to endosulfan. J Ind Pollut Control.

[CR8] Mwangala FS, Mundia PM, Nondo JC, Banda R, Mangoye C (1997) Persistence of lindane and endosulfan under field conditions in Zambia. Organochlor Insectic Afr Agroecosyst. 28(12):213–218

[CR44] Napit MK (2013). The effect of pesticides on fish fauna of Bhopal lower lake (MP). Afr J Environ Sci Technol.

[CR45] Ndimele P, Jenyo-Oni A, Kumolu-Johnson C, Chukwuka K, Onuoha S (2015). Effect of acute exposure to endosulfan (organochlorine pesticide) on hematology of African Mud Catfish, *Clarias gariepinus* (Burchell, 1822). Bull Environ Contam Toxicol.

[CR46] Nyangababo J, Henry L, Omutange E (2005). Organochlorine pesticide contamination in surface water, sediment, and air precipitation of Lake Victoria Basin, East Africa. Bull Environ Contam Toxicol.

[CR47] Ogbeide O, Tongo I, Ezemonye L (2015). Risk assessment of agricultural pesticides in water, sediment, and fish from Owan River, Edo State, Nigeria. Environ Monit Assess.

[CR48] Oliveira-Filho E, Geraldino B, Grisolia C, Paumgartten F (2005). Acute toxicity of endosulfan, nonylphenol ethoxylate, and ethanol to different life stages of the freshwater snail *Biomphalaria tenagophila* (Orbigny, 1835). Bull Environ Contam Toxicol.

[CR49] Otludil B, Cengiz EI, Yildirim MZ, Ünver Ö, Ünlü E (2004). The effects of endosulfan on the great ramshorn snail *Planorbarius corneus* (Gastropoda, Pulmonata): a histopathological study. Chemosphere.

[CR51] Pereira VM, Bortolotto JW, Kist LW, de Azevedo MB, Fritsch RS, da Luz Oliveira R (2012). Endosulfan exposure inhibits brain AChE activity and impairs swimming performance in adult zebrafish (Danio rerio). Neurotoxicology.

[CR52] Quinn L, de Vos B, Fernandes-Whaley M, Roos C, Bowman H, Kylin H, Pieters R, van den Berg J (2011) Pesticide use in South Africa: one of the largest importers of pesticides in Africa. In: Stoytcheva M (ed) Pesticides in the modern world—Pesticide use and management. InTech, pp 459–507

[CR53] Relyea R, Hoverman J (2006). Assessing the ecology in ecotoxicology: a review and synthesis in freshwater systems. Ecol Lett.

[CR54] Rozman KK, Klaassen CD (2007). Casarett and Doull’s toxicology: the basic science of poisons.

[CR55] Rubach MN, Crum SJ, Van den Brink PJ (2011). Variability in the dynamics of mortality and immobility responses of freshwater arthropods exposed to chlorpyrifos. Arch Environ Contam Toxicol.

[CR56] Schäfer RB, van den Brink PJ, Liess M, Sanchez-Bayo F, van den Brink PJ, Mann RM (2011). Impacts of pesticides on freshwater ecosystems. Ecological impacts of toxic chemicals.

[CR57] Stockholm Convention on Persistent Organic Pollutants Review Committee (2010) Report of the Persistent Organic Pollutants Review Committee on the work of its first meeting. UNEP/POPS/POPRC:6/13/Add.11, Geneva, Switzerland

[CR58] Syakalima M, Choongo K, Mwenechanya R, Wepener V, Yamasaki M, Maede Y (2006). Pesticide/herbicide pollutants in the Kafue river and a preliminary investigation into their biological effect through catalase levels in fish. Jpn J Vet Res.

[CR59] Tripathi G, Verma P (2004). Endosulfan-mediated biochemical changes in the freshwater fish *Clarias batrachus*. Biomed Environ Sci.

[CR60] United States Environmental Protection Agency (2011). Usage: 2006 and 2007 market estimates.

[CR61] van der Werf HM (1996). Assessing the impact of pesticides on the environment. Agric Ecosyst Environ.

[CR62] Van Dyk JS, Pletschke B (2011). Review on the use of enzymes for the detection of organochlorine, organophosphate and carbamate pesticides in the environment. Chemosphere.

[CR63] Weis JS, Candelmo A (2012). Pollutants and fish predator/prey behavior: a review of laboratory and field approaches. Curr Zool.

[CR64] World Health Organization (2000) Poisons information monograph 576. Chemical: Endosulfan. International Programme on Chemical Safety, Geneva, Switzerland

[CR65] Wurts WA, Durborow RM (1992). Interactions of pH, carbon dioxide, alkalinity and hardness in fish ponds.

[CR66] Yasser A, Naser M, Aziz N (2008). Acute toxicity of endosulfan to immature and adult gastropods Lymnaea radix cor (Annandale and Prashad, 1919). J Thi Qar Sci.

[CR67] Yekeen TA, Fawole OO (2011). Toxic effects of endosulfan on haematological and biochemical indices of *Clarias gariepinus*. Afr J Biotechnol.

[CR68] Zeid MA, Eldin I, Syed MA, Ramli J, Arshad JH, Omar I (2005). Bioaccumulation of carbofuran and endosulfan in the African Catfish *Clarias gariepinus*. Pertanika J Sci Technol.

