# Supplementary material for: Effects of Endosulfan on Predator–Prey Interactions Between Catfish and Schistosoma Host Snails
Source: Arch Environ Contam Toxicol. 2016 Mar 31;71:257–66. doi: 10.1007/s00244-016-0275-7 (PMC4935736; doi:10.1007/s00244-016-0275-7)
Supplement: Supplementary file 1 — Supplementary material 1 (DOCX 86 kb) [file 244_2016_275_MOESM1_ESM.docx]

**Supplementary Information**


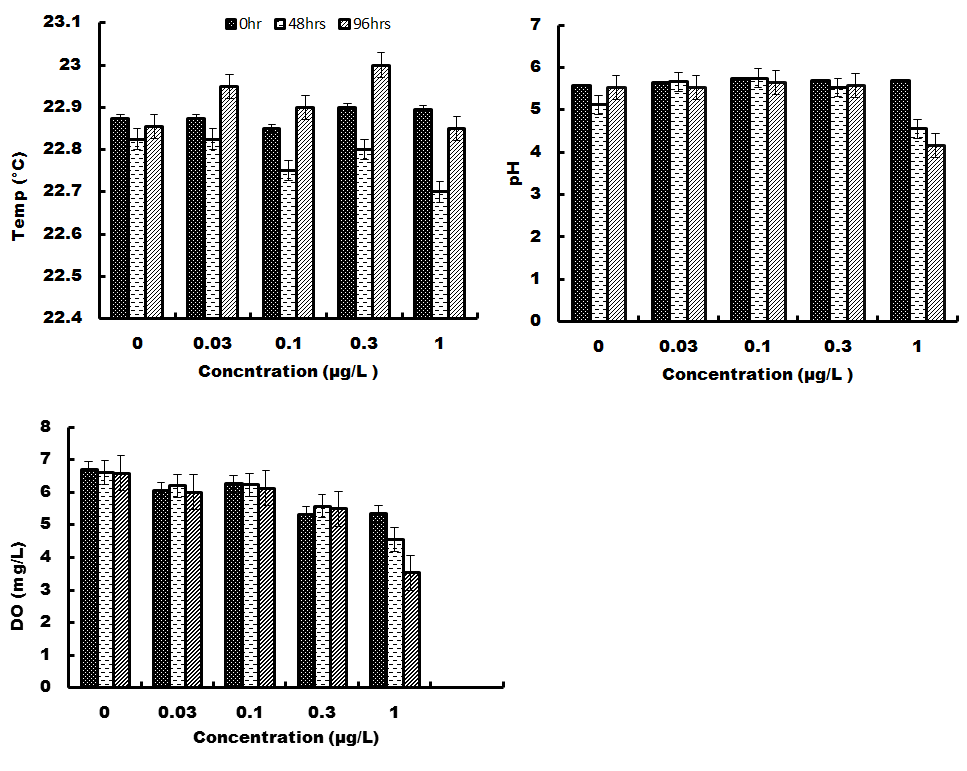


**Fig S1**: Temperature, pH and DO values at the beginning, halfway and at the end of the predation experiment at different concentrations of endosulfan.

Table S1: Endosulfan concentration in water and sediment recorded for water bodies in some African countries.

| **Location** | **Aquatic compartment** | | **Source** |
| --- | --- | --- | --- |
|  | **Water (μg/L)** | **Sediment (μg/Kg)** |  |
| Western Cape (Elgin farm dams) | 626 | - | (Ansara-Ross et al. 2012) |
| KwaZulu-Natal (Ubombo and Ingwavuma districts) | - | 0.09 – 2.36 | (Ansara-Ross et al. 2012) |
| Western Cape (Hex River valley) | 1.79 | - | (Ansara-Ross et al. 2012) |
| Western Cape (Lourens River) | 0.03 – 0.16 | 3.9 – 245 | (Ansara-Ross et al. 2012) |
| Western Cape (Hex River valley, Grabouw and Piketberg) | <0.10 – 26.3 | - | (Ansara-Ross et al. 2012) |
| Eastern Cape (East London and Umtata) | <0.02 – 0.10 | <bdl – 92.0 | (Ansara-Ross et al. 2012) |
| Gauteng (Jukskei River) | 7.28 –364 | 44.7 – 3 705 | (Ansara-Ross et al. 2012) |
| Western Cape (Berg and Franschhoek rivers) | 0.01 | 4.60 – 156 | (Ansara-Ross et al. 2012) |
| Warri River (Nigeria) | 0.01 - 9.23 | 0.06 – 11.98 | (Ezemonye et al. 2010) |
| Ogbese River ( Nigeria) | bdl^a^  <bdl – 6.17^b^  3.01 – 10.9^c^ | 25.6 – 199  8.35 – 69.4  4.69 – 63.6 | (Ibigbami et al. 2015) |
| El Rahaway drain (Egypt) | 0.021 – 0.823 |  | (El Bouraie et al. 2011) |
| Lake Volta (Ghana) | 0.039 | 1.3 | (Gbeddy et al. 2015) |
| Ghana | 0.062^a^  0.031^b^  0.031^c^ | 0.19  0.13  0.23 | (Ntow 2001) |
| Lake Victoria basin (East Africa) | 0.003 - 0.034^a^  0.012 – 0.031^b^  0.017 – 0.038^c^ | 0.12 – 0.43^a^  0.39 – 0.45^b^  0.27 – 0.42^c^ | (Nyangababo et al. 2005) |
| Kafue River (Zambia) | - | 3.00 | (Syakalima et al. 2006) |

NB: bdl = below detection limit, a = alpha-endosulfan, b = beta endosulfan, c = endosulfan sulfate

Table S2: Observed behavioural responses for catfish exposed to various concentrations of endosulfan.

| Response | Concentration | | | | | | | | | | | | | | | | | | | |
| --- | --- | --- | --- | --- | --- | --- | --- | --- | --- | --- | --- | --- | --- | --- | --- | --- | --- | --- | --- | --- |
|  | 0.0µg/L | | | | 0.5µg/L | | | | 1.0µg/L | | | | 1.1µg/L | | | | 1.2µg/L | | | |
|  | R 1 | R2 | R 3 | R 4 | R 1 | R 2 | R 3 | R 4 | R 1 | R 2 | R 3 | R 4 | R 1 | R2 | R 3 | R 4 | R 1 | R 2 | R 3 | R4 |
| Swimming | - | - | - | - | +++ | +++ | +++ | +++ | +++ | +++ | +++ | +++ | + | + | + | + | + | + | + | + |
| Jumping | - | - | - | - | +++ | +++ | +++ | +++ | ++ | ++ | ++ | ++ | + | + | + | + | + | + | + | + |
| Gasping | - | - | - | - | ++ | ++ | ++ | ++ | +++ | +++ | +++ | +++ | +++ | +++ | +++ | +++ | +++ | +++ | +++ | +++ |
| Disorientation | - | - | - | - | ++ | ++ | ++ | ++ | ++ | ++ | ++ | ++ | +++ | +++ | +++ | +++ | +++ | +++ | +++ | +++ |
| Mucus | - | - | - | - | ++ | ++ | ++ | ++ | +++ | +++ | +++ | +++ | +++ | +++ | +++ | +++ | +++ | +++ | +++ | +++ |
| Resting | - | - | - | - | + | + | + | + | + | + | + | + | +++ | +++ | +++ | +++ | +++ | +++ | +++ | +++ |

(-) = normal, (+) = low, (++) = moderate, (+++) = severe

**Table S3**: Catfish survival at each concentration over time in the toxicity experiment with endosulfan. In all replicates 5 individuals were included at the start of the experiment. At 72 hours all fish were dead at all concentrations except the control.

| Conc. (µg/L) | 24 h | | | | | 48 h | | | | |
| --- | --- | --- | --- | --- | --- | --- | --- | --- | --- | --- |
|  | R 1 | R 2 | R 3 | R 4 | % surv | R 1 | R 2 | R 3 | R 4 | % surv |
| 0 | 5 | 5 | 5 | 4 | 95 | 5 | 5 | 5 | 4 | 95 |
| 0.5 | 5 | 5 | 4 | 5 | 95 | 4 | 5 | 4 | 5 | 90 |
| 1.0 | 3 | 3 | 2 | 3 | 55 | 2 | 3 | 2 | 2 | 45 |
| 1.1 | 0 | 1 | 0 | 0 | 5 | 0 | 0 | 0 | 0 | 0 |
| 1.2 | 0 | 0 | 0 | 0 | 0 | 0 | 0 | 0 | 0 | 0 |

N.B: R = Replicate

**Table S4**: *B. globosus* survival at each concentration over time in the toxicity test with endosulfan. In all replicates 20 individuals were included at the start of the experiment.

| Conc. (µg/L) | 24 h | | | | | 48 h | | | | | 72 h | | | | | 96 h | | | | |
| --- | --- | --- | --- | --- | --- | --- | --- | --- | --- | --- | --- | --- | --- | --- | --- | --- | --- | --- | --- | --- |
|  | R1 | R2 | R3 | R4 | %  surv | R1 | R2 | R3 | R4 | % surv | R1 | R2 | R3 | R 4 | % surv | R 1 | R 2 | R 3 | R 4 | % surv |
| 0 | 19 | 20 | 20 | 20 | 98.7 | 19 | 20 | 17 | 20 | 81 | 17 | 20 | 17 | 20 | 79 | 15 | 15 | 13 | 15 | 61.2 |
| 100 | 20 | 20 | 20 | 20 | 100 | 20 | 18 | 19 | 20 | 82 | 17 | 17 | 18 | 20 | 77 | 15 | 17 | 17 | 14 | 70.2 |
| 200 | 19 | 17 | 19 | 20 | 80 | 19 | 14 | 14 | 16 | 67 | 18 | 14 | 14 | 16 | 66 | 10 | 14 | 14 | 16 | 58 |
| 400 | 20 | 19 | 20 | 20 | 89 | 20 | 19 | 17 | 19 | 79.7 | 18 | 12 | 17 | 19 | 70.5 | 15 | 10 | 9 | 13 | 58.7 |
| 500 | 18 | 18 | 19 | 20 | 80 | 16 | 17 | 16 | 18 | 71.5 | 14 | 13 | 15 | 16 | 62 | 14 | 11 | 15 | 16 | 60 |
| 1000 | 18 | 12 | 17 | 16 | 67 | 10 | 10 | 14 | 13 | 58.7 | 6 | 7 | 9 | 9 | 38.7 | 2 | 5 | 5 | 4 | 20 |
| 1200 | 17 | 18 | 17 | 19 | 75.7 | 7 | 9 | 10 | 5 | 38.7 | 5 | 7 | 7 | 4 | 28.7 | 0 | 3 | 6 | 0 | 11.2 |

N.B: R = Replicate

Table S5: Daily residual snails for all treatments of the predation experiment. Replicates (R) in which the predator died and was replaced by a new individual are indicated in bold.

| Time | Concentration | | | | | | | | | | | | | | | | | | | |
| --- | --- | --- | --- | --- | --- | --- | --- | --- | --- | --- | --- | --- | --- | --- | --- | --- | --- | --- | --- | --- |
|  | 0.0µg/L | | | | 0.03µg/L | | | | 0.1µg/L | | | | 0.3µg/L | | | | 1.0µg/L | | | |
|  | R 1 | R2 | R 3 | R 4 | R 1 | R 2 | R 3 | R 4 | R 1 | R 2 | R 3 | R 4 | R 1 | R2 | R 3 | R 4 | R 1 | R 2 | R 3 | R4 |
| 0 hrs | 50 | 50 | 50 | 50 | 50 | 50 | 50 | 50 | 50 | 50 | 50 | 50 | 50 | 50 | 50 | 50 | 50 | 50 | 50 | 50 |
| 24 hrs | 35 | 33 | 32 | 36 | 39 | 36 | 40 | 40 | 43 | 44 | 44 | 43 | 46 | 45 | 45 | 44 | 49 | **50** | 50 | **50** |
| 48 hrs | 14 | 11 | 15 | 18 | 32 | 31 | 35 | 34 | 36 | 34 | 36 | 33 | 34 | **38** | 36 | 37 | **49** | 46 | **44** | 47 |
| 72 hrs | 5 | 9 | 11 | 10 | 21 | 26 | 24 | 28 | 27 | 29 | 29 | 33 | 30 | 30 | 33 | 34 | 47 | **46** | **43** | 47 |
| 96 hrs | 1 | 4 | 4 | 2 | 16 | 23 | 19 | 24 | 22 | 25 | 27 | 29 | 27 | 28 | 31 | 27 | 44 | 44 | 43 | 45 |
